# Cases of Cancer Treated with Cundurango

**Published:** 1871

**Authors:** D. W. Bliss

**Affiliations:** Washington, D. C., Professor of Urinary Pathology in the Medical Department of Georgetown College


III.—*Cases of Cancer Treated with Cundurango.* By D. W. Bliss, M.D., of Washington, D. C., Professor of Urinary Pathology in the Medical Department of Georgetown College.

---

My attention was first attracted to this remarkable agent during a professional attendance upon Mr. Flores, the Minister from Ecuador, through whom his government had conveyed to our Secretary of State a portion of the shrub, together with printed statements of its successful employment by eminent South American physicians. Having always conceived that a remedy would sooner or later be discovered for the cure of carcinoma and tuberculosis, so long the *opprobria medicinæ,* I was at once interested in the direct and encouraging exposition of the Quito doctors. With the hope of benefitting my own patients, and effecting some good for others, I determined promptly to test its merits by actual experiment, regardless of the charges and possible opposition to which I knew my honest efforts would be subjected by the hypercritical and ungenerous of my professional *confreres.* Fortunately, several cases of unequivocal carcinoma were then under treatment. Accustomed to the remorseless ravages of a mallady for which even the surgeon's knife afforded no adequate relief, I approached the experiment not without misgivings of success, but with the fixed purpose to render the test as complete as the limited supply of the plant in my possession would allow.

Mrs. Matthews, the mother of Hon. Schuyler Colfax, had been the victim of mammary cancer for a long period, which had already assumed secondary and constitutional symptoms in a marked degree. On the 29th of April last, I placed her on the decoction of cundurango, and had the gratification of observing an early and decided change for the better, in both the local and general conditions. One of its almost immediate effects was the relief of pain, and a free diaphoresis, characterized by an odor distinctly observable of the infusion itself. Upon the return of Mrs. Matthews to her place of residence in Indiana, I still continued to direct her treatment, and furnished the requisite supplies of the medicine.

On the 9th of May, just thirteen days after the commencement of the new remedy, her husband addressed me a letter, from which I make the following extracts:

"The stony condition of the tumor has given place to softness. This morning I notice about one-third of the surface has turned from a scarlet to a white color, and it has commenced suppurating as though the thing were dead and coming out. The whole tumor is very much flattened, the discharge is different and not near so offensive. The greatest improvement is in her complexion. From a *tallowy,* puffy-looking, and somewhat bluish skin, she is regaining her old natural look, the skin shrinking, becoming wrinkled and clear.

"I am so happy in the prospect of a cure that I feel like a new man, as though a ton of lead had been lifted from my heart. Is it not a little singular, it has not had any perceptible effect on her nervous system? Her digestion is good, and she begins to feel that she will get well."

On the 14th of the same month Mr. Matthews writes as follows:

"This is the seventeenth day since I commenced the use of cundurango; shall cease for a few days, and note carefully the effect. When I began the treatment, Mrs. Matthews' breast was almost as hard as a stone, about four inches in diameter, the cancer itself two inches in diameter, with raised edges, hard and scarlet-colored, bleeding profusely at the slightest touch, emitting an odor of the most sickening and disagreeable kind, discharging a brownish, cancerous, limpid fluid; the countenance bloated, tallowy-looking, with a bluish pallor of the whole face; the lips turned blue at the least exertion, so that I have been very much alarmed, fearing a rapid crisis and dissolution; at the same time the tumor itself enlarged with fearful rapidity, so much so that I could notice the growth from day to day.

"Now all is changed—the countenance has resumed its old, familiar look; she moves about with great sprightliness, the blue of the lips no longer indicating fatigue or effort. The granular swelling under the chin is gone; strength increasing; the tumor itself much flattened and decreased in protuberance; the color changed to a white, maturating sore; the limpid, cancerous discharge ceased, and in its place a healthy discharge of white matter much less offensive; the hardened glands are soft to the touch, the whole symptoms indicating most plainly to me that the treatment has, so far, neutralized the poison of the blood, and that another short campaign with cundurango will insure a complete cure."

On the 2d of the present month I visited Mrs. Matthews, at South Bend, and was indeed astonished at the rapid change which had taken place. The tumor had become soft, the color natural, the secondary glandular deposits had all disappeared. The improved complexion, muscular firmness, and elasticity of spirits, all pointed to an early and complete recovery.

Mrs. Handy, residing on M street in this city, was the next subject of experiment with the cundurango. This was a highly-typical and fearfully-advanced case of cancer uteri. The grayish color, unequal, irregular elevations of the ulcer edges, the sympathetic disturbance of the bladder, the paroxysms of intese pain, together with the hot, dry, shrivelled, yellow surface, the wasted muscles, sunken eyes, the small, quick, wiry pulse, revealed one of those sad cases, where all hope of remedy fails.

The cundurango, in the form of decoction, was administered first to Mrs. Handy on the 31st day of last month. A regular record has been kept from day to day, describing the least change of symptoms, but I have not the space to introduce it here. Suffice it that even in this extreme case the beneficial effects of this wonderful remedial agent have been most apparent. The pain has steadily declined, the diseased parts are less tumefied and sensitive, and the discharge is very slightly offensive. The cachectic appearance of this patient has much improved, and she expresses herself as feeling altogether better.*

A lady of the family of Hon. Mr. Gorham, Secretary of the

*It is to be regretted that the supply of cundurango for this patient was exhausted a week ago, and no more will be in hand until the 1st proximo.

United States Senate, has had mammary cancer of several months' duration, and her condition was pronounced hopeless by leading Northern surgeons. I was called to see her on the 1st of June, of this year, and found cancer of the breast, with secondary deposits in the shoulder and humeral portion of the left arm, attended by extreme rigidity of the neck, and almost complete immobility of the affected limb.

A careful daily record has been preserved of this case also, by which the most decided improvement is indicated. The mammary tumor has grown softer, and the line of skin-attachment bisecting the nipple is much less marked. The head, before stiff, is now perfectly free and movable, while the natural mobility of the disabled arm is restored, and the tissues, before hard, are now soft and natural. The general condition progresses favorably *pari passu* with the local improvement.

To both of these last-mentioned cases I have invited my experienced professional friend, Dr. C. C. Cox, and the history of the treatment and its results have been carefully observed by that eminent physician. It may be proper to state that letters have been pouring in upon me from persons at a distance, suffering from cancer, who have had the opportunity to use but a very small portion of the remedy, and yet who report marvellous improvement in all the symptoms. As the readers of your *Journal* may not have seen it, I shall give here the results of a chemical analysis of the plant made by Prof. Antisell, of this city:

ANALYSIS.

*Physical Description.*—Stem woody, shrubby, and covered with greenish or ash-gray bark, the former tint being due to a coating of lichens on the surface. The branches are from half an inch to little more than an inch in diameter, the average being about the thickness of the finger. The woody fibre is straw-colored and brittle, breaking with a sharp fracture; it is almost tasteless, slightly aromatic, and bitter. *Bark.*—This contains whatever medicinal virtues are in the plant. It is of a gray color, slightly ribbed or fluted longitudinally from corrugation, the result of drying; it increases in thickness in the ratio of the increase of the stem—in the thicker branches constituting more than half the weight of the whole, in the thinner somewhat less than half; readily separable from the stem by pounding or bruising, when it comes off in clean, longitudinal pieces; brittle in the transverse fracture, having a warm, camphory, aromatic, and bitter taste, resembling the cascarilla of the older collections. Under the lens it is readily resolved into three layers: 1. The inner layer or cambium of reticular woody tissues, having granules of starch, and particles of resin embedded. 1. A middle layer of woody fibre and dotted ducts, with resinous particles also in this layer. 3. The cuticular or outer layer of bark-cells, of a brown color, and containing tannic acid and coloring matters.

Ratio of wood and bark, average of three examinations:

*Chemical Agalysis of Bark.*

| | |
|---|---|
| Bark | 49.72 |
| Wood | 50.28 |
| Total | 100.00 |

Constitution of bark in 100 parts:

| | |
|---|---|
| Moisture | 8 |
| Mineral salts | 12 |
| Vegetable matters | 80 |
| Total | 100 |

These vegetable matters were separable, by the usual methods, into the following:

| | |
|---|---|
| Fatty matter, soluble in ether and partially in strong alcohol | .7 |
| Yellow resin, soluble in alcohol | 2.7 |
| Starch, gum, and glucose | .5 |
| Tannin, yellow and brown coloring matter, and extractive | 12.6 |
| Cellulose, lignin, etc | 64.5 |
| Total | 80. |

On distillation, no volatile oil or acid was obtainable.

No crystalline alkaloid, or active principle, was separable by the usual method of proximate analysis.

Whatever medicinal virtues the plant may possess must reside either in the yellow resin or in the extractive. The former is soluble in alcohol, the latter in water. In the water-decoction some of the resin is diffused, but the greater portion of resin is not extracted by water.

The therapeutic position of the plant, judged from analysis, is among the aromatic bitters.

I regret I am compelled to leave this interesting subject for the present. As facts accumulate hereafter, they will be promptly given to the profession, and my earnest efforts are pledged to extend to the suffering as rapidly as possible the advantage of this new and surprising agent of the materia medica.—*N. Y. Medical Journal.*

